# Supplementary material for: Folding of a bacterial integral outer membrane protein is initiated in the periplasm
Source: Nat Commun. 2017 Nov 3;8:1309. doi: 10.1038/s41467-017-01246-4 (PMC5670179; doi:10.1038/s41467-017-01246-4)
Supplement: Supplementary file 1 — Supplementary Information [file 41467_2017_1246_MOESM1_ESM.pdf]

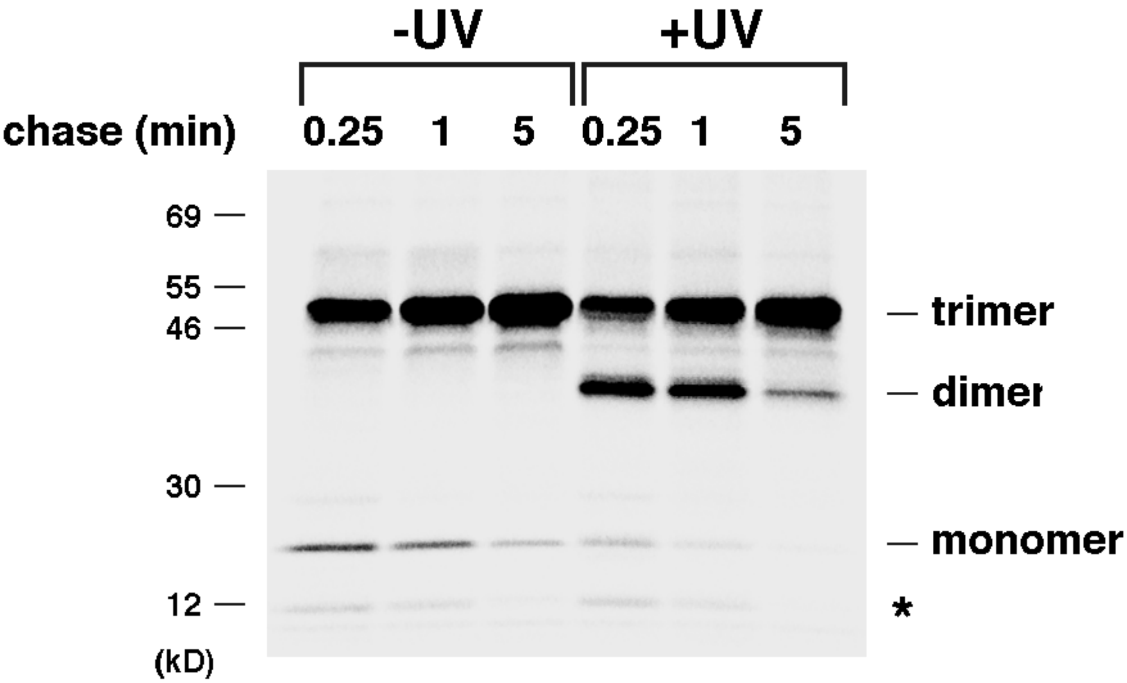

Supplementary Figure 1. UpaGΔ2 does not form a crosslink to Skp. HDB131 (AD202 Δ*skp*; see ref. 1) transformed with pRS4 [P_{trc}-*HA-upaGΔ2(Y1735am)*] and pDULE-Bpa were subjected to pulse-chase labeling. Half of the cells were UV irradiated, and immunoprecipitations were performed using an anti-HA antiserum. The truncated form of the protein that resulted from translation termination at the amber codon is denoted with an asterisk.

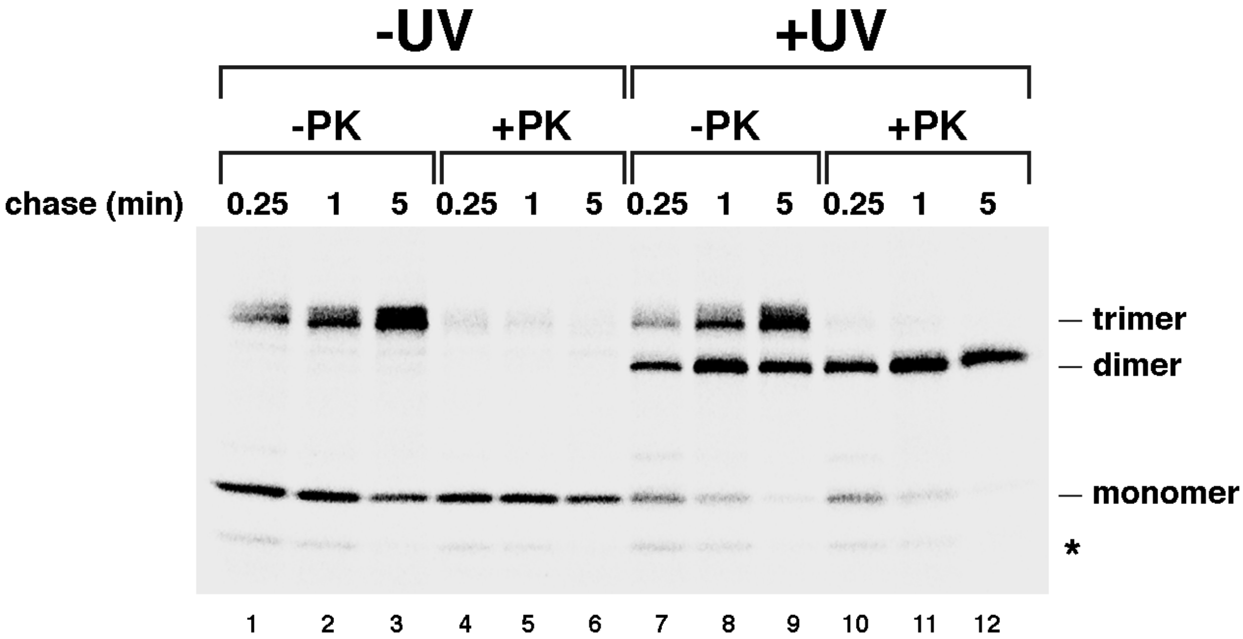

Supplementary Figure 2. Incubation at low temperature increases the half-life of the UpaGΔ2 crosslinking product. The experiment shown in Fig. 2B was repeated, except that the culture was shifted to 25° C before radiolabeling.

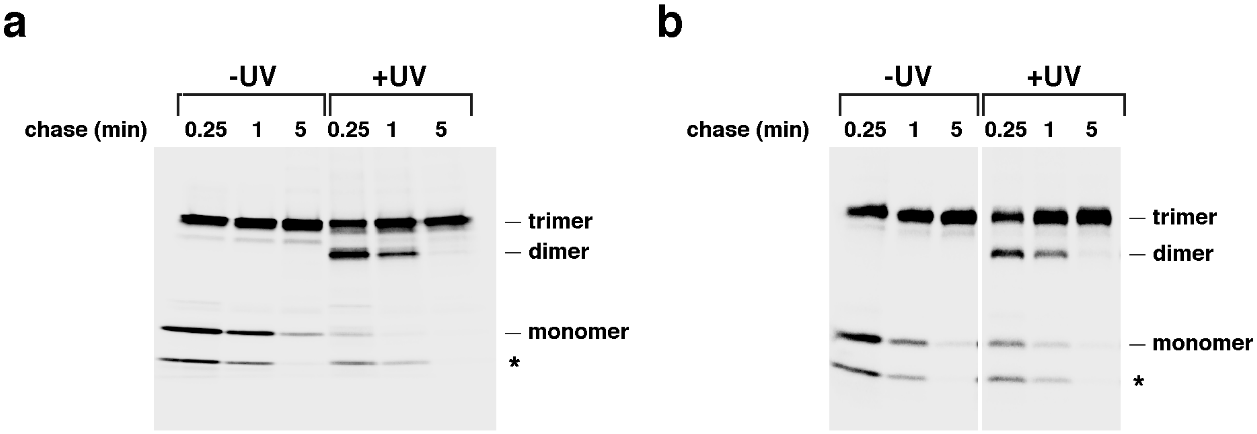

Supplementary Figure 3. His_{10}- and FLAG-tagged versions of UpaGΔ2 assemble rapidly. AD202 transformed with pDULE-Bpa and either pRS5 [P_{trc}-*His_{10}-upaGΔ2(Y1735am)*] **(a)** or pRS6 [P_{trc}-*FLAG-upaGΔ2(Y1735am)*] **(b)** were subjected to pulse-chase labeling. After half of the cells were UV irradiated, His-tagged proteins were isolated on NiNTA beads and FLAG-tagged proteins were immunoprecipitated using an anti-FLAG antiserum. The truncated form of the protein that resulted from translation termination at the amber codon is denoted with an asterisk.

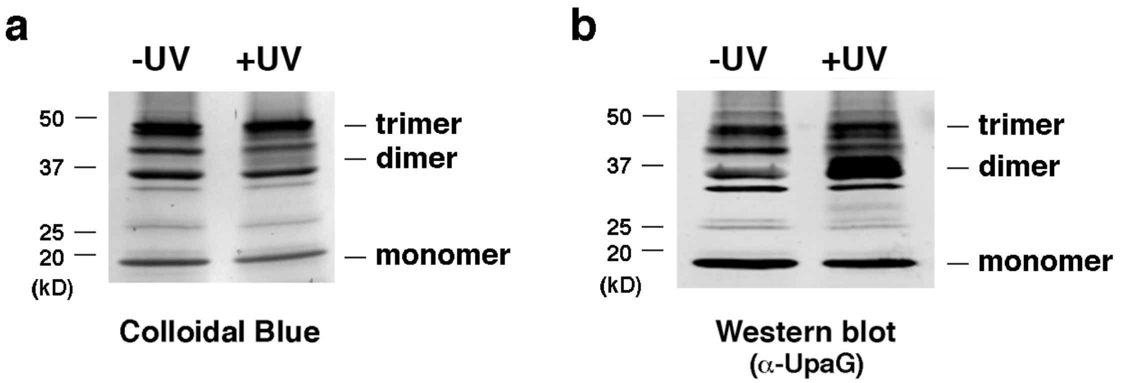

Supplementary Figure 4. Purification of the UpaGΔ2 crosslinking product. AD202 were transformed with pRS5 [P_{trc}-*His$_{10}$-upaGΔ2 (Y1735am)*], and half of the cells were UV irradiated. His_{10}-tagged proteins were purified from cell membranes of irradiated and non-irradiated cells by NiNTA chromatography and resolved by SDS-PAGE. **(a)** A portion of the gel following Colloidal Blue staining is shown. A faint ~38 kD band that is observed only in the +UV sample likely corresponds to the crosslinking product. **(b)** A portion of the NiNTA eluates was analyzed by Western blot using an anti-UpaG antiserum.

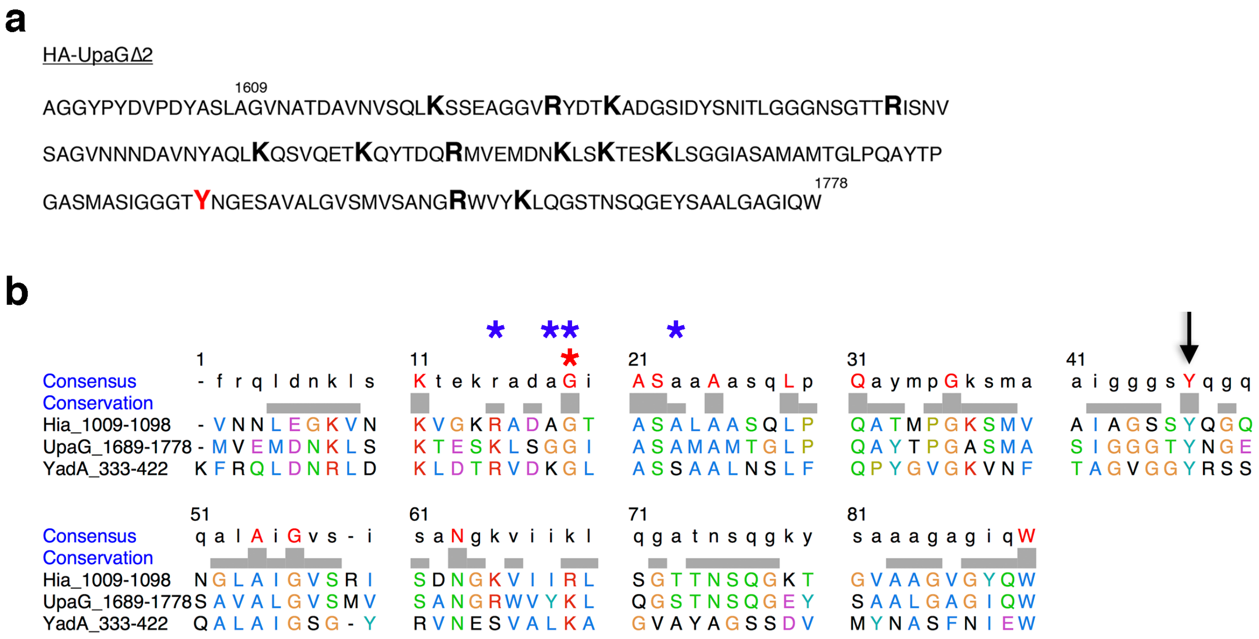

Supplementary Figure 5. Amino acid sequence of HA-UpaGΔ2 and alignment of TAA β barrel domains. **(a)** The amino acid sequence of HA-UpaGΔ2 is shown. Basic residues (bold) and Y1735 (red) are highlighted. **(b)** The C-terminal 90 residues of Hia, UpaG and YadA were aligned using Clustal Omega (http://www.ebi.ac.uk/Tools/msa/clustalo/). The conserved tyrosine that corresponds to Y1735 in UpaG is denoted with an arrow. Amino acids in the adjacent subunit of Hia and YadA that are located within 4 Å of the conserved tyrosine and that are situated in the region that corresponds to the large tryptic fragment of UpaGΔ2 (plus the upstream lysine residue) are denoted with red and blue asterisks, respectively. The formation of a crosslink to the lysine residue would likely prevent access to trypsin.

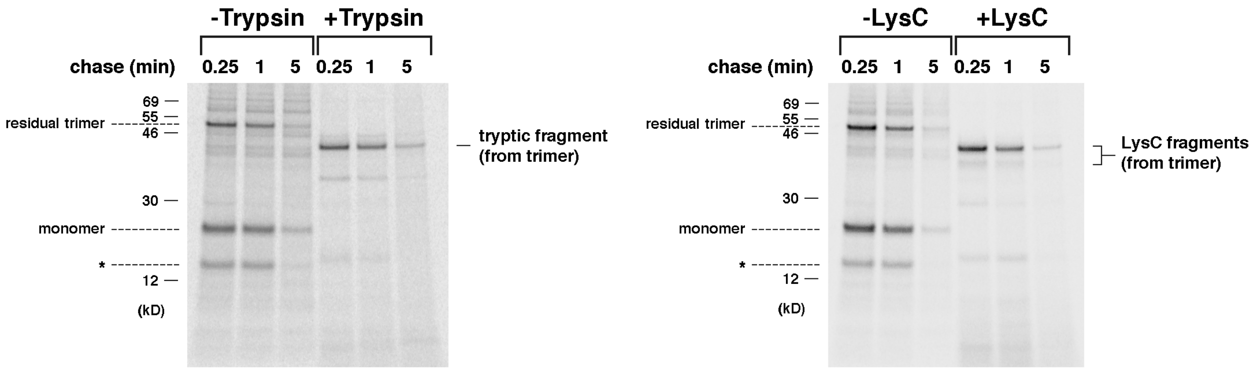

Supplementary Figure 6. Digestion of UpaGΔ2 containing Bpa at position 1735 does not yield ~12 kD tryptic and Lys-C fragments in the absence of UV light. Control samples that were not UV irradiated in the experiment shown in Fig. 3C were analyzed by SDS-PAGE.

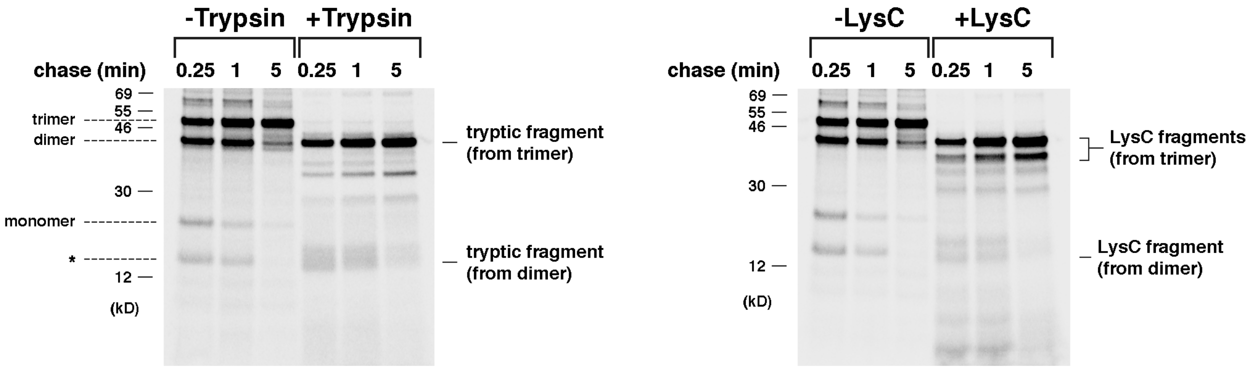

Supplementary Figure 7. The stable UpaGΔ2 trimer yields predominantly high molecular weight tryptic and LysC fragments. Control samples that were not treated with PK in the experiment shown in Fig. 3C were analyzed by SDS-PAGE.

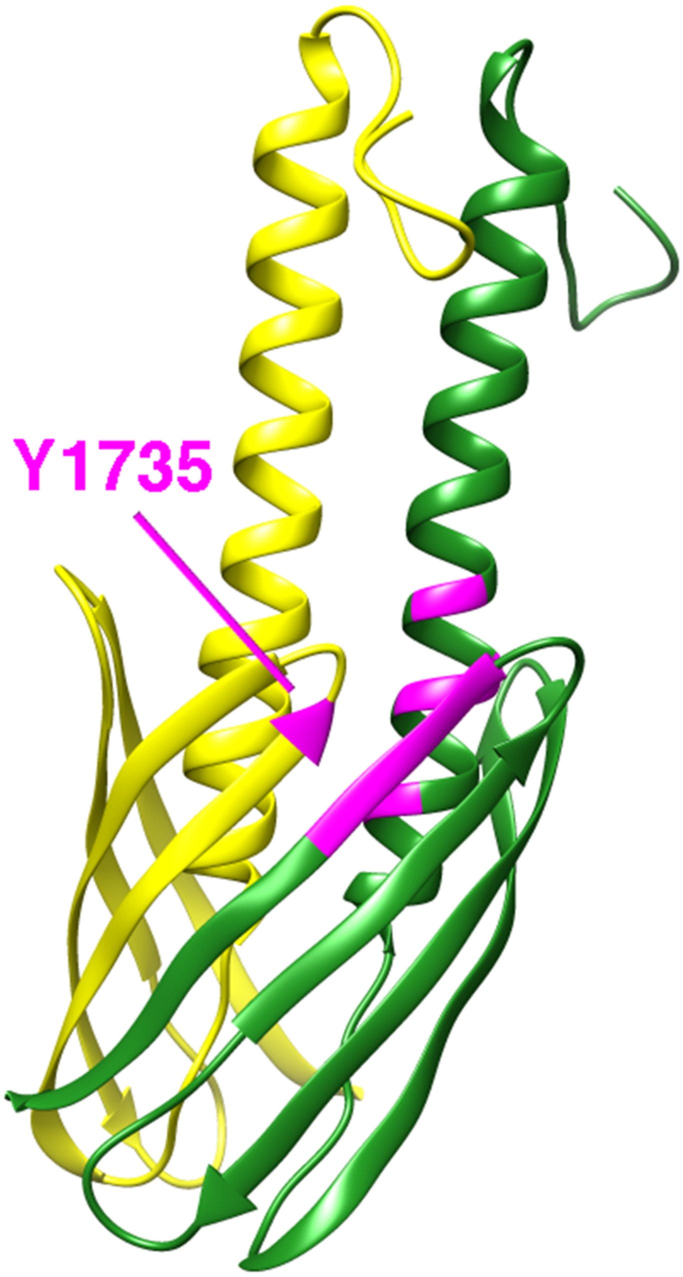

Supplementary Figure 8. Structure of two subunits of YadA visualized using UCSF Chimera software. The conserved tyrosine (Y380) that is equivalent to UpaG Y1735 in one subunit and all of the residues located within 4 Å in the adjacent subunit are shown in magenta. The structural information was derived from the NMR structure of the fully folded YadA β barrel domain[2].

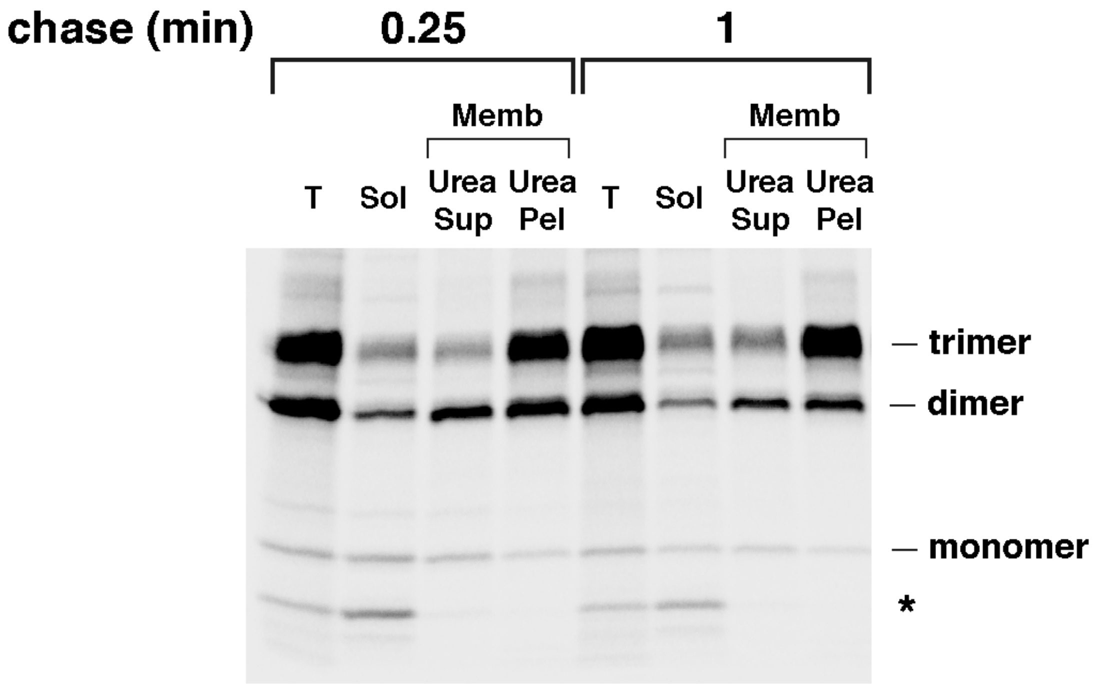

Supplementary Figure 9. A darker exposure of the gel in Fig. 5 is shown.

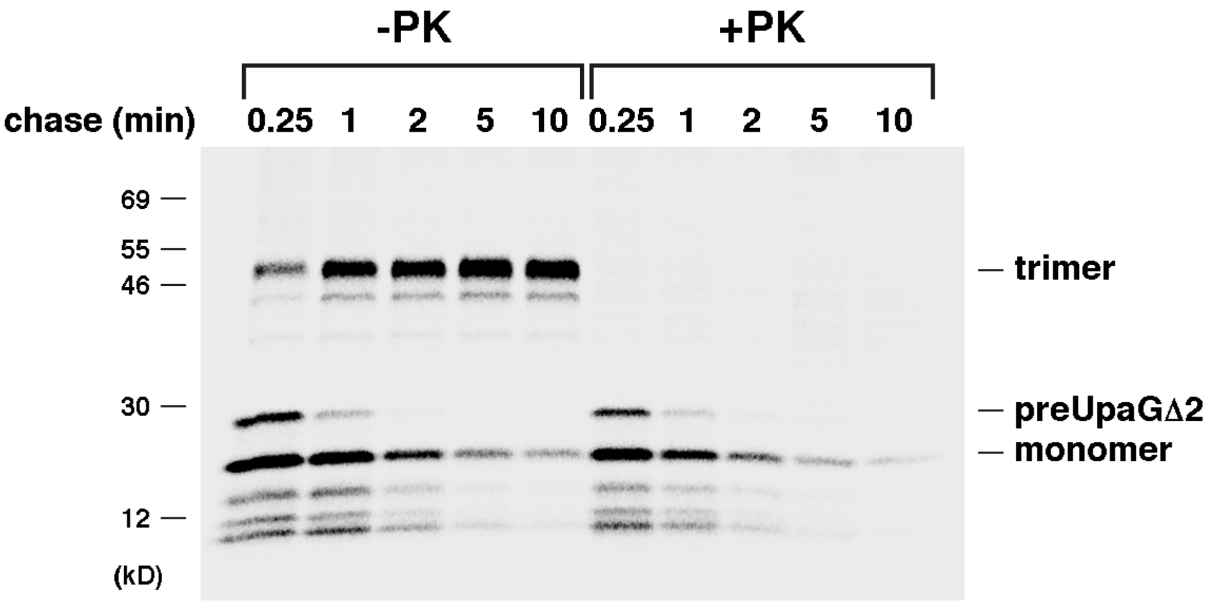

Supplementary Figure 10. Assembly of a targeting-deficient UpaGΔ2 mutant under slow growth conditions. AD202 transformed with pRS7 [P_{trc}-*HA-upaGΔ2(W1778A)*] were grown in M9 medium and subjected to pulse-chase labeling. Half of the cells were treated with PK, and immunoprecipitations were then performed using an anti-HA antiserum.

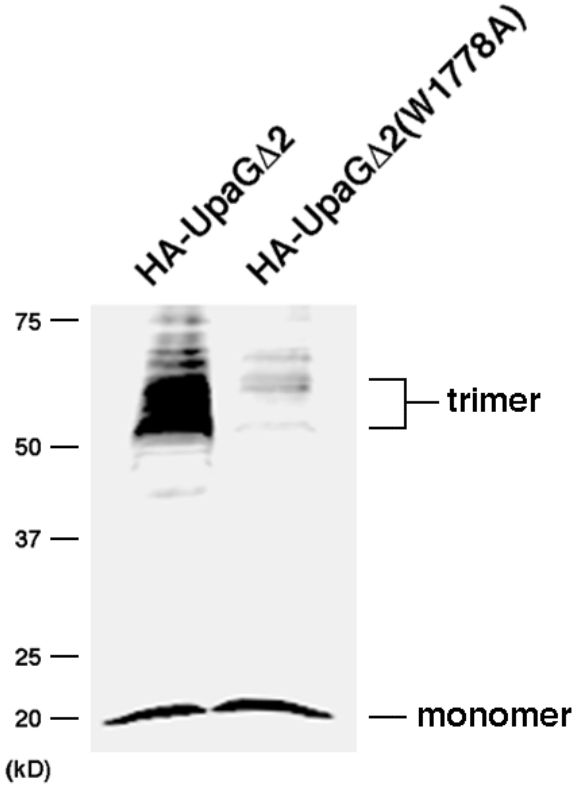

Supplementary Figure 11. A targeting-deficient UpaGΔ2 mutant does not assemble in rich medium. AD202 transformed with pRS1 [P_{trc}-*HA-upaGΔ2*] or pRS7 [P_{trc}-*HA-upaGΔ2(W1778A)*] were grown in LB, and 10 μM IPTG was added at OD_{600}=0.6. After 30 min cells were lysed with Bug Buster Master Mix, and equivalent amounts of each cell lysate (normalized by OD_{600}) were analyzed by Western blot using an anti-HA antiserum.

Supplementary Table 1: PCR primers used in this study

Name	Sequence (Restriction sites underlined)
PRS1001	5’-GCGCGCGAATTCATGGCGTATTTTGGATGATAACGAGGCGCAACATATGAACAAAATATTTAAAGTTATCTGG-3’ (EcoRI)
PRS1002	5’-TATATACGGCCGATGCCCCAAACGACGACAACAAC-3’ (EagI)
PRS1003	5’-GTACTTAGAACGGTGAATCGGCTGTTG-3’
PRS1004	5’-CCGTTCTAAGTACCGCCACCAATAGAG-3’
PRS1005	5’-GAGAGACATATGGCCGGTGTTAATGCC-3’ (NdeI)
PRS1006	5’-GCGCGCGGATCCTTACCACTGAATACCGGCAC-3’ (BamHI)
PRS1007	5’-GTGTGTTCTAGATCGGCCGCAGGTGGTGACTACAAAGACGATGACGACAAGCTGGCCGGTGTTAATGCCACC-3’ (XbaI)
PRS1008	5’-ACAGCCAAGCTTAGCGCATCAGGCAATGTG-3’ (HindIII)
PRS1009	5’-GCTGGCGAATTCATGGCGTATTTTGGATG-3’ (EcoRI)
PRS1010	5’-GCAAGCGAATTCAGCGCATCAGGCAATGTG-3’ (EcoRI)
PRS1011	5’-GCTGGCAAGCTTGAATTCATGGCGTATTTTG-3’ (HindIII)
PRS1012	5’-GGTATTCAGGCGTAATCATCCATTAACAAATGG-3’
PRS1013	5’-GGATGATTACGCCTGAATACCGGCACC-3’
PRS1014	5’-GCGCGCAAGCTTTCAGCAGGATCACATATGAACAAAATATTTAAAG-3’ (HindIII)

SUPPLEMENTARY REFERENCES

1. Ieva, R. & Bernstein, H.D. Interaction of an autotransporter passenger domain with BamA during its translocation across the bacterial outer membrane. *Proc. Natl. Acad. Sci. USA* **106**, 19120-19125 (2009).
2. Shahid, S.A. *et al*. Membrane-protein structure determination by solid-state NMR spectroscopy of microcrystals. *Nat. Methods* **9**, 1212-1217 (2012).
