# Supplementary material for: Folding of a bacterial integral outer membrane protein is initiated in the periplasm
Source: Nat Commun. 2017 Nov 3;8:1309. doi: 10.1038/s41467-017-01246-4 (PMC5670179; doi:10.1038/s41467-017-01246-4)
Supplement: Supplementary file 3 — Description of Additional Supplementary Files [file 41467_2017_1246_MOESM3_ESM.pdf]

Description of Additional Supplementary Files

File Name: Supplementary Data 1
Description: Peptides identified in analysis of 36-40 kD polypeptides by mass spectrometry

File Name: Supplementary Data 2
Description: Proteins identified in analysis of 36-40 kD polypeptides by mass spectrometry
